# Correlation between tear fluid and serum vitamin D levels

**DOI:** 10.1186/s40662-016-0053-7

**Published:** 2016-09-02

**Authors:** Swaminathan Sethu, Rohit Shetty, Kalyani Deshpande, Natasha Pahuja, Nandini Chinnappaiah, Aarti Agarwal, Anupam Sharma, Arkasubhra Ghosh

**Affiliations:** 1 GROW Research Laboratory, Narayana Nethralaya Foundation, #258/A Hosur Road, Narayana Health City, Bommasandra, 560099 Bangalore, India; 2 Cornea and Refractive Surgery Division, Narayana Nethralaya, Bangalore, India

**Keywords:** Vitamin D, 25-hydroxyvitamin D, 1,25-dihydroxyvitamin D, Tears, Serum

## Abstract

**Background:**

Vitamin D deficiency is associated with a range of systemic diseases including ocular disorders. The objective of this study is to measure tear vitamin D levels and investigate the correlation between serum and tear vitamin D levels.

**Methods:**

A total of 48 healthy volunteers without any systemic and ocular disease were recruited for this observational cohort study. Serum was collected using clot activator coated Vacutainer® Plus tubes. Tear fluid was collected using Schirmer’s strips. Serum and tear total 25-hydroxyvitamin D levels were measured by competitive chemiluminescent ELISA and the correlation between the levels were studied.

**Results:**

The measured serum 25-hydroxyvitamin D level ranged between 3.3 and 27.5 ng/ml (Mean ± SEM, 9.4 ± 0.7 ng/ml; Median 8.4 ng/ml). Significantly (*p* < 0.0001) higher level of 25-hydroxyvitamin D was detected in the tears (Mean ± SEM, 17.0 ± 1.6 ng/ml; Range 3.2–45.8 ng/ml; Median, 16.3 ng/ml) compared to serum. An average of ~2 fold (Mean ± SEM, 1.9 ± 0.2; Range 0.4–5.8; Median, 1.7) higher 25-hydroxyvitamin D was observed in tears compared to serum in the subjects. In addition, a positive correlation was observed between serum and tear 25-hydroxyvitamin D levels (*r* = 0.5595; *p* < 0.0001).

**Conclusions:**

A higher level of 25-hydroxyvitamin D was observed in the tear fluid compared to that of the serum. It would be beneficial to consider tear vitamin D levels to study its role with reference to ocular surface diseases.

## Précis

25-hydroxyvitamin D measured in the tear fluid was found to be higher than in the corresponding serum sample.

## Main text

### Background

Vitamin D is well known for regulating calcium homeostasis, immune response, cellular proliferation and differentiation, angiogenesis, apoptosis and nociception [1, 2]. A high prevalence of vitamin D deficiency has been documented worldwide [1]. The optimal vitamin D (total 25-hydroxyvitamin D) level ranges between 30 and 80 ng/ml in the serum, and deficiency/insufficiency of vitamin D is considered when total 25-hydroxyvitamin D is <20 ng/ml [3, 4]. Owing to its pleiotropic nature and ubiquitous expression of vitamin D receptor (VDR) in almost all cells and tissues [5], deficiency of vitamin D has been associated with a range of diseases, and supplementation of vitamin D has substantially improved the prognosis of these diseases [6]. Vitamin D status in the serum is suitable for studying its association with systemic conditions. However, it would be beneficial to obtain tissue specific vitamin D status for localized conditions such as ocular surface disease. There is increasing evidence regarding extra-renal synthesis and tissue-specific effects of vitamin D3 in other tissues [7], including, the eye [8–11]. The cells and tissues in the eye are responsive to 1,25-dihydroxyvitamin D since VDR is present in the epithelium of the cornea, lens and ciliary body, corneal endothelium, retinal pigment epithelium, ganglion cell layer and photoreceptors of the human eye [8, 12]. Hence, vitamin D status is being associated with the incidence and severity of various ocular conditions, including myopia, age-related macular degeneration, diabetic retinopathy and uveitis [10]. Serum 25-hydroxyvitamin D levels were also reported to be associated with some ocular surface conditions such as dry eye and allergic conjunctivitis [13–15]. In vivo reports provide evidence concerning the anti-inflammatory and immunomodulatory roles of vitamin D in the corneal region [16, 17]. The role of vitamin D in ocular surface conditions, mechanistic insights into the aetiopathology of vitamin D deficiency and beneficial effects of vitamin D supplementation in the management of ocular surface conditions are yet to be studied. Determining local vitamin D levels in the eye is essential to meet this knowledge gap. Therefore, the current study aims to quantify 25-hydroxyvitamin D in tear fluid and correlate its level with that in the serum.

### Methods

#### Study design & clinical examination

The observational cohort study approved by the Narayana Nethralaya Institutional Review Board (Ref. No.: C/2015/05/05) was conducted in adherence to Indian Council for Medical Research (ICMR) guidelines and tenets of the Declaration of Helsinki. A total of 48 healthy volunteers were selected (after obtaining informed written consent) for the study after thorough clinical investigation at Narayana Nethralaya Eye Hospital, Bangalore, India to rule out any ongoing or recent ocular and/or systemic co-morbidity. In addition, contact lens wearers were not included in the study.

#### Serum and tear sample collection

Serum was isolated by centrifuging peripheral venous blood collected in BD Vacutainer® Plus Plastic Serum Tubes (BD, New Jersey, USA) with spray-coated silica as a clot activator and stored in −80 °C until further use. Tear fluid from the subjects were collected using sterile Schirmer’s strip (5 × 35-mm^2^; Tear Strips, ContacareOpthalmics and Diagnostics, India) by following Schirmer’s Test I procedure and stored at -80 °C in a sterile microcentrifuge tube until further use. Tear fluid was extracted from Schirmer’s strips by agitating small cut pieces of these strips in phosphate buffered saline (PBS) solution in a sterile microcentrifuge tube at +4 °C for 1.5 h. Tear fluid was then eluted by centrifugation and stored at −80 °C until further use. Schirmer’s strip based tear fluid collection was followed as it was reported to be suitable and comparable with capillary tube based tear fluid collection for downstream analysis [18].

#### Measurement of serum and tear vitamin D

Total 25-hydroxyvitamin D (25-hydroxyvitamin D_3_ + 25-hydroxyvitamin D_2_) levels in the serum and tear fluid were measured by direct competitive chemiluminescent enzyme linked immunoassay – 25-hydroxyvitamin D ELISA Kit (Enzo Life Sciences, Switzerland). The kit was optimized and validated to detect serum vitamin D. As assays to detect tear 25-hydroxyvitamin D are unavailable, we adapted the above mentioned kit to quantify tear 25-hydroxyvitamin D. Since the tear fluid was eluted in PBS, we included additional PBS based 25-hydroxyvitamin D_3_ (Cayman Chemical, Ann Arbor, MI, USA) standards for the assays. 25-hydroxyvitamin D_3_ is the most predominant and endogenous form of vitamin D which is the major contributor towards measured total vitamin D - 25-hydroxyvitamin D levels. The accuracy of this modified assay results was evaluated by comparing results from an automated direct competitive chemiluminescent enzyme linked immunoassay (ADVIA Centaur® Vitamin D Total, Siemens) that detects both 25-hydroxyvitamin vitamins D_2_ and D_3_. The latter was chosen to validate our method because it was reported to have acceptable accuracy compared with LC-MS/MS [19]. Furthermore, the levels of tear 25-hydroxyvitamin D were normalized to the amount of tear fluid collected for the measurements.

#### Statistical analysis

All statistical analyses were performed with GraphPad Prism 6.0 (GraphPad Software, Inc., La Jolla, CA, USA) and Stata 12.1 (StataCorp, Texas USA). Shapiro-Wilk normality test was used to check the distribution of the data set. Spearman correlations analysis, Wilcoxon matched-pairs signed rank test and Mann-Whitney test were used to analyse data sets that were not normally distributed. The agreement between the data sets was analysed using the Bland-Altman plot. The mean or median value of the individual groups was reported as Mean ± SEM or median (along with the range). Two-tailed *p* < 0.05 was considered to be statistically significant.

### Results

**Fig. 1:**
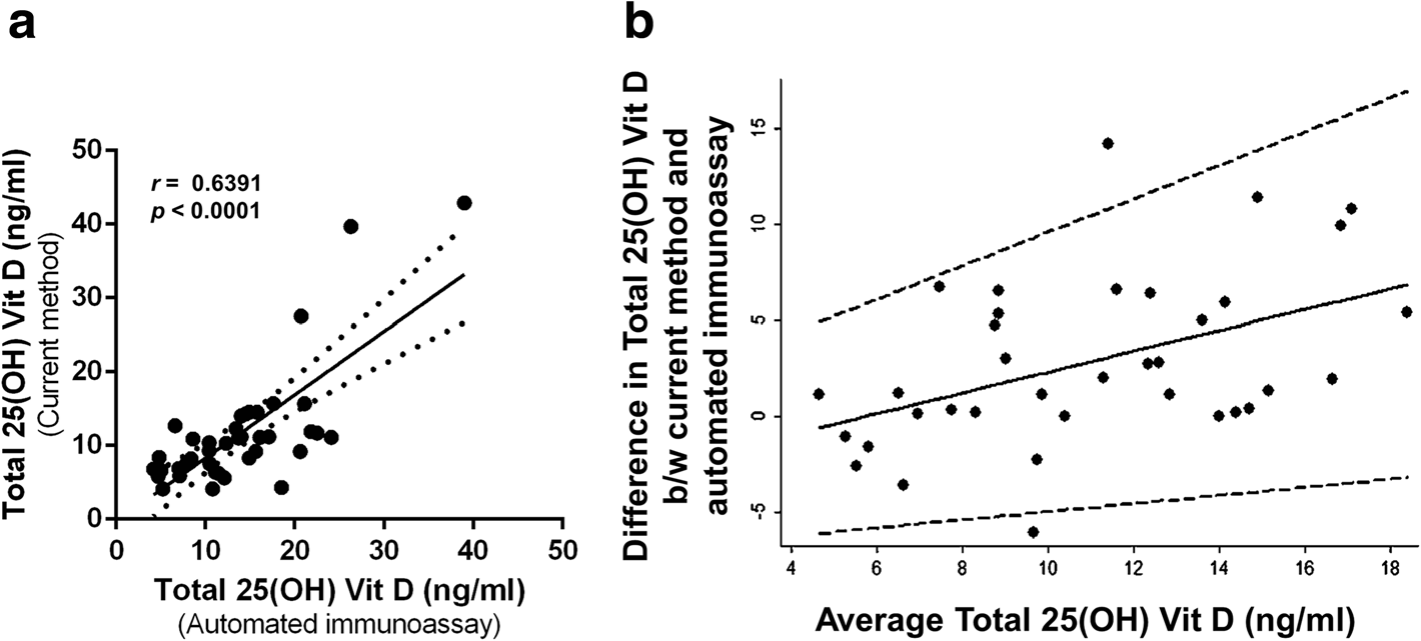
Comparative analysis of serum 25-hydroxyvitamin D measurement protocols. **a** Graph shows the correlation of serum total 25-hydroxyvitamin D [25(OH) Vit D] levels measured by manual direct competitive chemiluminescent enzyme linked immunoassay with PBS-based 25-hydroxyvitamin D_3_ standards; current method (*y axis*) and automated direct competitive chemiluminescent enzyme linked immunoassay (*x axis*). *r* - Spearman correlation; *n* = 36. **b** Bland-Altman plot showing the agreement between the two methods used to measure serum 25-hydroxyvitamin D. The average total 25-hydroxyvitamin D in the *x*-axis indicates the mean of serum 25-hydroxyvitamin D level measured by the current method using PBS-based 25-hydroxyvitamin D_3_ standards and by automated immunoassay

The study cohort included 21 male and 27 female subjects of Indian origin. The age of subjects in the cohort ranged from 19 to 53 years (Mean ± SEM, 31 ± 1.1 years; Median 28.5 years). To evaluate the validity of our modified method in estimating total 25-hydroxyvitamin D in serum and tears, the levels of 25-hydroxyvitamin D in serum were measured in 36 samples using modified method and automated immunoassay. A significant positive correlation (*r* = 0.6391, Spearman correlation; *p* < 0.0001; *n* = 36) was observed between the two methods (Fig. 1a). The Bland-Altman plot evaluating the agreement between serum 25-hydroxyvitamin D levels measured by these methods showed that 95 % limits of agreement were between -5.8 and 11.4 ng/ml (Fig. 1b). These observations support the applicability of the modified method in measuring 25-hydroxyvitamin D in both serum and tears.

**Fig. 2:**
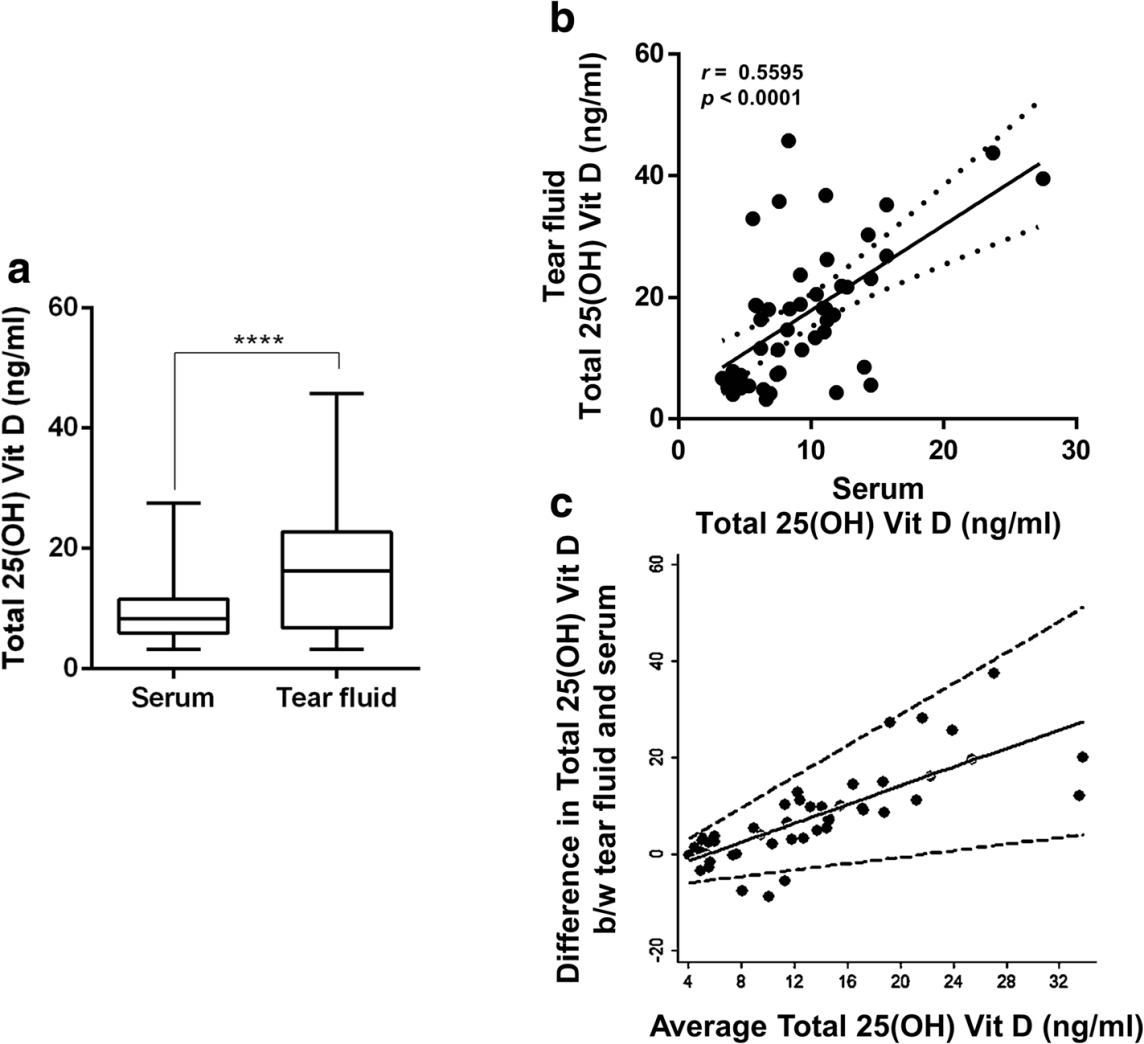
Correlative evaluation of total 25-hydroxyvitamin D levels in serum and tear fluid. **a** Box and whiskers plot indicating the median levels of total 25-hydroxyvitamin D measured in the serum and tear fluid. **b** Correlation between serum and tear total 25-hydroxyvitamin D [25(OH) Vit D] levels (*n* = 48), *r* - Spearman correlation. **c** Bland-Altman plot showing the agreement between tear and serum 25-hydroxyvitamin D levels. The average total 25-hydroxyvitamin D in the *x*-axis indicates the mean of tear and serum 25-hydroxyvitamin D levels. *n* = 48; ****p < 0.0001, Wilcoxon matched-pairs signed rank test

The measured serum 25-hydroxyvitamin D level in 48 subjects ranged between 3.3 and 27.5 ng/ml (Mean ± SEM, 9.4 ± 0.7 ng/ml; Median, 8.4 ng/ml). A total of 64.6 % (31/48) of the study cohort had serum 25-hydroxyvitamin D <10 ng/ml (severe deficiency), 31.3 % (15/48) had serum 25-hydroxyvitamin D between 11 and 20 ng/ml (moderate deficiency) and 4.2 % (2/48) was between 21 and 30 ng/ml (vitamin D insufficiency). No significant difference was observed in the serum 25-hydroxyvitamin D level between male (Mean ± SEM, 9.1 ± 0.8 ng/ml; Median, 9.3 ng/ml) and female (Mean ± SEM, 9.7 ± 1.1 ng/ml; Median, 7.6 ng/ml) subjects in the study cohort. The tear 25-hydroxyvitamin D level was 17.0 ± 1.6 ng/ml (Mean ± SEM) with values ranging between 3.2 and 45.8 ng/ml (Median, 16.3 ng/ml). Similar to serum 25-hydroxyvitamin D, significant difference was not observed in the tear 25-hydroxyvitamin D level between male (Mean ± SEM, 15.7 ± 2.5 ng/ml; Median, 13.4 ng/ml) and female (Mean ± SEM, 18.1 ± 2.2 ng/ml; Median, 1.8 ng/ml) subjects in the study cohort. However, a significantly (*p* < 0.0001) higher tear 25-hydroxyvitamin D compared to matched serum 25-hydroxyvitamin D levels was observed (Fig. 2a). Post-hoc calculation determined that the power of detection was greater than 80 % and hence the sample size was adequate for the observation made. 35.4 % (17/48) of subjects had tear 25-hydroxyvitamin D <10 ng/ml, 35.4 % between 11 and 20 ng/ml, 12.5 % (6/48) between 21 and 29 ng/ml and 16.6 % (8/48) with >30 ng/ml. A significant positive correlation (*r* = 0.5595, Spearman correlation; *p* < 0.0001) was also observed between tear and serum 25-hydroxyvitamin D (Fig. 2b). The Bland-Altman plot evaluating the agreement between tear and serum 25-hydroxyvitamin D levels shows 95 % limits of agreement were between -10.78 and 26.06 ng/ml (Fig. 2c). Tear 25-hydroxyvitamin D levels were higher than serum in the majority of the subjects. It was found to be 1.9 ± 0.2 (Mean ± SEM) fold higher than the serum 25-hydroxyvitamin D levels when quantified in the current modified method. The fold difference ranged between 0.4 and 5.8 (Median, 1.7). In 16.7 % (8/48) of subjects the tear 25-hydroxyvitamin D was lower than that of its matched serum levels and in 83.3 % (40/48) of samples the tear 25-hydroxyvitamin D was either equal or higher than that measured in the respective matched serum. Among the samples in which the tear 25-hydroxyvitamin D was either equal or higher than serum, in 20 % (8/40) the difference was within 2 ng/ml, in 45 % (18/40) the difference was >2 but ≤10 ng/ml, in 25 % (10/40) the difference ranged between 10 and 20 ng/ml and in 10 % (4/40) the difference was between 21 and 37.5 ng/ml. The current observations suggest that tear fluid may have a higher level of 25-hydroxyvitamin D compared with serum.

### Discussion

Serum vitamin D deficiency is associated with various systemic conditions and recently its relevance in ocular health and disease is being reported [10]. There is growing evidence regarding 25-hydroxyvitamin D status in ocular surface diseases such as allergic conjunctivitis and dry eye [13, 14, 20]. However, there has been some rather contradicting observations with regards to 25-hydroxyvitamin D and dry eye [15, 21]. Nevertheless, all these reports have been based on serum 25-hydroxyvitamin D. We suggest that assessing 25-hydroxyvitamin D status in tears would hold more relevance in eye disease, especially in ocular surface conditions. Vitamin D (both 25-hydroxyvitamin D_3_ and 1,25-dihydroxyvitamin D_3_) was shown to influence corneal epithelial barrier function by regulating expression of occludin [11], and the ability of corneal epithelial cells to synthesize and metabolize vitamin D has also been documented [8, 9]. An in vivo study in rabbits showed that 25-hydroxyvitamin D can be measured in tears and its level increases in the tears following oral supplementation of vitamin D [9]. In the current study, we have shown that 25-hydroxyvitamin D can be measured in human tears by competitive chemiluminescent immunoassay and that the 25-hydroxyvitamin D levels were significantly higher in the tears than in the serum. Similarly, another study reported significantly higher 25-hydroxyvitamin D levels in tears (71.8 ± 6.2 ng/ml) compared to serum (21.8 ± 11.3 ng/ml) in children (12.5 ± 2.5 years) using electro chemiluminescent immunoassay [22]. The difference in the 25-hydroxyvitamin D levels between tear fluid and serum seem to vary between the current study and that reported by Goksugur SB et al. The primary reason underlying this difference could be related to the principle and sensitivity of the assay used to measure 25-hydroxyvitamin D [23, 24]. Moreover, we have modified the method to render more accurate measurement of 25-hydroxyvitamin D in tear fluid. The age difference between these study cohorts could also be considered as a possible factor contributing to the variation. It should also be noted that contrary to our observation and that of Goksugur SB et al., 25-hydroxyvitamin D levels in tears was lower than plasma in rabbits [9], this could once again be attributed to the technique adopted for quantifying 25-hydroxyvitamin D and possible inter-species differences. Since the human corneal epithelium is capable of synthesizing vitamin D [9], we speculate that the increased level observed in the tears could be due to vitamin D produced by the corneal epithelium following exposure of the eye to UVB rays present in sunlight. Unlike tear fluid, 25-hydroxyvitamin D in the saliva (another non-invasive source for measuring vitamin D) was reported to be manifold lower than that of the serum [25, 26]. Hence, more detailed studies are required to confirm increased 25-hydroxyvitamin D in tear fluid and understand the source that contributes to it.

### Conclusion

The observations from the current study suggest that 25-hydroxyvitamin D is present in tear fluid and its level was found to be higher than in the corresponding serum in humans. It would be beneficial if studies investigating the association between tear vitamin D and ocular surface disease can determine its relevance with reference to disease severity and pathogenesis so as to inform the need for topical supplementation of vitamin D to ameliorate ocular surface diseases.
